# Exudative versus Nonexudative Age-Related Macular Degeneration: Physiopathology and Treatment Options

**DOI:** 10.3390/ijms23052592

**Published:** 2022-02-26

**Authors:** Ana Rita Fernandes, Aleksandra Zielińska, Elena Sanchez-Lopez, Tiago dos Santos, Maria Luisa Garcia, Amelia M. Silva, Jacek Karczewski, Eliana B. Souto

**Affiliations:** 1 i3s—Institute for Research and Innovation in Health, University of Porto, R. Alfredo Allen 208, 4200-135 Porto, Portugal; anaritavfernandes@gmail.com (A.R.F.); tiago.f.santos@ineb.up.pt (T.d.S.); 2 INEB—Biomedical Engineering Institute, University of Porto, Alfredo Allen 208, 4200-135 Porto, Portugal; 3 FEUP—Faculty of Engineering, University of Porto, R. Dr. Roberto Frias, 4200-465 Porto, Portugal; 4 Department of Pharmacy, Pharmaceutical Technology and Physical Chemistry, Faculty of Pharmacy, University of Barcelona, 08001 Barcelona, Spain; esanchezlopez@ub.edu (E.S.-L.); marisagarcia@ub.edu (M.L.G.); 5 Centre for Research and Technology of Agro-Environmental and Biological Sciences, CITAB, UTAD, Quinta de Prados, 5001-801 Vila Real, Portugal; amsilva@utad.pt; 6 Institute of Human Genetics, Polish Academy of Sciences, Strzeszyńska 32, 60-479 Poznań, Poland; aleksandra.zielinska@igcz.poznan.pl; 7 Institute of Nanoscience and Nanotechnology (IN2UB), University of Barcelona, 08001 Barcelona, Spain; 8 Department of Biology and Environment, University of Trás-os-Montes e Alto Douro, UTAD, Quinta de Prados, 5001-801 Vila Real, Portugal; 9 Department of Environmental Medicine, Poznan University of Medical Sciences, 60-479 Poznań, Poland; 10 Department of Gastroenterology, Dietetics and Internal Diseases, H. Swiecicki University Hospital, Poznan University of Medical Sciences, 60-355 Poznań, Poland; 11 Department of Pharmaceutical Technology, Faculty of Pharmacy, University of Porto, Rua de Jorge Viterbo Ferreira, 228, 4050-313 Porto, Portugal; 12 CEB—Centre of Biological Engineering, University of Minho, Campus de Gualtar, 4710-057 Braga, Portugal; 13 LABBELS—Associate Laboratory, 4800-122 Braga, Portugal

**Keywords:** age-related macular degeneration, inflammatory cascade, antiangiogenic agents, choroidal neovascularization, geographic atrophy

## Abstract

Age-related macular degeneration (AMD) is an eye disease typically associated with the aging and can be classified into two types—namely, the exudative and the nonexudative AMD. Currently available treatments for exudative AMD use intravitreal injections, which are associated with high risk of infection that can lead to endophthalmitis, while no successful treatments yet exist for the nonexudative form of AMD. In addition to the pharmacologic therapies administered by intravitreal injection already approved by the Food and Drug Administration (FDA) in exudative AMD, there are some laser treatments approved that can be used in combination with the pharmacological therapies. In this review, we discuss the latest developments of treatment options for AMD. Relevant literature available from 1993 was used, which included original articles and reviews available in PubMed database and also information collected from Clinical Trials Gov website using “age-related macular degeneration” and “antiangiogenic therapies” as keywords. The clinical trials search was limited to ongoing trials from 2015 to date.

## 1. Introduction

Age-related macular degeneration (AMD) is considered a multifactorial disease, with environmental and genetic elements as the main risk factors [1]. AMD is one of the most common causes of irreversible vision loss in patients over 65 years old. The eye is a complex organ that is connected to the brain, and the brain is responsible for the interpretation of information collected by the eye. There are about 170 million people affected with AMD worldwide, which makes this disease the third leading blinding cause in the world [2,3]. AMD is responsible for approximately 9% of the cases of blindness worldwide [4]. Aging does contribute significantly to AMD; in fact, the age-adjusted prevalence is approximately 24% in people aged from 65 to 74 and more than 44% in those aged from 70 to 95 [5,6]. Patients with AMD have a decrease in quality of life since several daily routine activities require functional central visual perception, such as driving and reading [7]. Frequently, AMD patients are diagnosed with depression and have frequent bone fractures due to recurrent falls and vision loss [8,9,10].

In addition to the risk factors described in Table 1, it is vital to know the family history of the patients—e.g., smoking habits and hypertension [11]. Some researchers showed that a diet rich in fats might increase the risk of developing AMD—i.e., diets with high values of fat and trans-fat have a significant risk of developing AMD compared with diets rich in omega-3 fatty acids and fish [12]. Some evidence suggests that lifestyle modifications are important in delaying the onset and progression of AMD [13]. The cessation of smoking and a high level of physical activities also demonstrated a decrease in the risk of progression of AMD [14].

The risk of developing AMD increases with changes in specific genes—namely, complement factor H (CFH) gene (at chromosome 1); complement factor B (CFB) gene; complement component 2, 3, and 5; and LOC gene (chromosome 10) [15,16,17,18]. The presence of single nucleotide polymorphisms in the CFH gene on chromosome 1 and genes (PLEKHA1 and LOC387715) on chromosome 10 that encode proteins involved in inflammatory cascades significantly increases the risk of developing AMD [16]. The polymorphisms in CFB are described to have protective effects [19]; the same happens with the polymorphisms of complement component 2 gene on chromosome 6 and other haplotypes on the CFH [20]. Linked to C3b, CFH protects the cells from damage induced by the complement. The CFH is an essential anti-inflammatory agent; thus, if the activity of the complement is abnormal, the complement cascade activation occurs and, consequently, the inflammatory response in the subretinal tissues [19]. Tobacco is a risk factor for the disease, as it decreases the level of CFH and, consequently, increases the risk of developing AMD when compared with the non-smokers with the CFH polymorphism [21,22]. The chronic inflammation could be decreased by the protective polymorphism of the CFB and the complement component 2 genes that are usually involved in the beginning of the complement cascade [19]. There are more than 30 proteins in the complement system, and they work as an inflammatory cascade, inducing phagocytosis, agglutination, and increased chemotaxis [23]. Inflammatory cytokines such as IL-6 (pro-angiogenic), IL-18 (pro-angiogenic), and tumor necrosis factor (TNF)-alpha (pro-inflammatory) are, as expected, increased in ocular tissues of patients with AMD [24,25,26].

With aging and due to the chronic low-grade in the levels of oxidative stress, the retinal innate immune system, as microglia and the complement system, experiences low levels of activation—a phenomenon called para-inflammation. Para-inflammation response is able to maintain the homeostasis in older healthy eyes. On the other hand, this para-inflammatory response is dysregulated in AMD patients, thus promoting the set-up of macular damage. Para-inflammation dysregulation (chronic inflammation) is influenced by genetic predisposition, age, and behavior risk factors [27]. Pro-inflammatory cytokines, such as IL-6, IL-8, and TNF-alpha stimulate macrophages, and the tissue complement system stimulates tissue repair, thus maintaining homeostasis, and consequently, restoration of functionality. The tissue factors released into circulation are responsible for stimulating the systemic immune system (systemic inflammation). The tissue stress initiates other innate immune response (complement pathway) to promote remodulation and tissue repair. The adaptative response originated by the innate immune system of affected tissues is the para-inflammation that helps the adaptation of tissue under stressful conditions and consequently restores its functionality [28].

**Table 1:** Relevant risk factors, prevalence, and prognosis of wet and dry age-related macular degeneration (AMD) (CNV, choroidal neovascularization).

	Race	Sex	Age	Prevalence	Prognosis	References
Exudative AMD	Caucasian people are far more likely to have wet AMD and vision loss compared with black people and Hispanics.	Women have an increased risk for AMD when compared with men.	There is a positive correlation between the prevalence and progression of the disease with aging.	In the United States, AMD occurs in 10% of the population aged between 65 and 74 years and in 25% of population with more than 74 years. 10% to 20% of people with nonexudative AMD progress to exudative disease.	The prognosis for exudative AMD is significantly worse when compared with nonexudative AMD.	[29,30,31]
Nonexudative AMD	Higher incidence in Caucasian people (especially in patients with light-colored eyes) compared with African Americans. Incidence in Asians is increasing.	Unknown difference between women and men.	The disease prevalence increases in each decade of life. More common in patients older than 70 years.	In the United States: -AMD is the principal cause of blindness in people older than 50 years. -In population older than 60 years the prevalence is greater than 20%. -Late atrophy and CNV have an incidence of 0% in patients with 50 years or less. In patients with 70 years the incidence is 2% and at 80 years the prevalence is 6%.	Better prognosis when compared with wet AMD. People with nonexudative AMD will have steady gradual deterioration of visual acuity. Is usual to have other visual dysfunctions, i.e., loss of contrast sensitivity.	[31,32,33,34]

**Figure 1:**
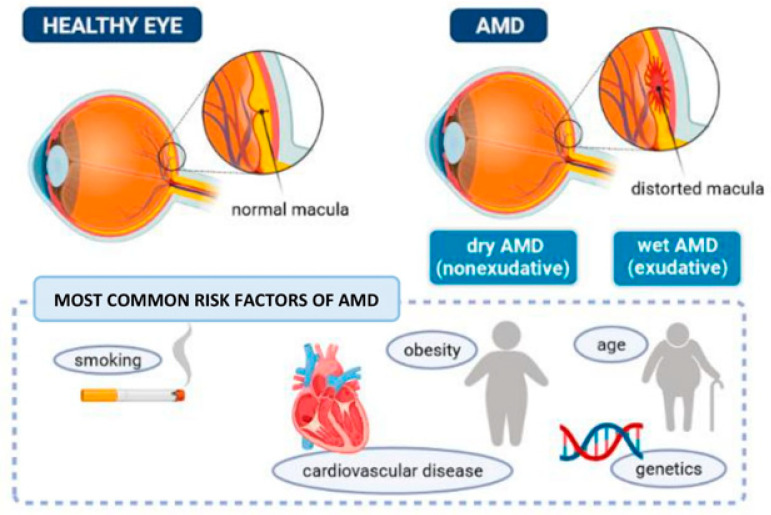
The eye with AMD and main factors increasing the risk of disease.

The prevalence of any form of AMD, either early-stage or late-stage, is reported to be higher in Europeans (16%) in comparison with Asians (9%) [35]. A meta-analysis of AMD prevalence published in 2014 projected an increase to 288 million cases worldwide in 2040, with a higher AMD burden to Europeans than Africans, Asians, and Hispanics [4]. AMD can be categorized into two types—namely, the nonexudative (or the dry AMD) and the exudative (or the wet AMD), as shown in Figure 1.

The basis of dry AMD is the accumulation of extracellular material under the retinal pigment epithelium (RPE). This deposition can lead to the formation of drusen. Drusen is composed of lipids, vitronectin, and inflammatory and amyloid proteins. The alterations present in dry AMD, both atrophic and hypertrophic, happen in the epithelium underlying the central macula and consequently promote the loss of the photoreceptors present in the retina. Patients with nonexudative AMD can progress to an exudative form of AMD [36]. The exudative form of AMD (wet AMD) is characterized by the formation of pathological choroidal neovascular membranes (CNM) under the retina, which can leak fluid and blood. CNM in the worst scenario can lead to a disciform scar affecting the central vision in a relatively short time, if not properly treated. Approximately 20% of patients with dry AMD will mostly progress to the wet form of AMD [37]. In AMD, oxidative stress is believed to be the major trigger in pathogenesis due to the combined exposure of the retina to oxygen and light. In addition to smoking habits, other factors that influence the development of AMD are a high level of body mass index (>25) and blue light exposure. Studies showed that exposure to blue light as emitted by smartphones and other devices caused vision damage and accelerated the risk to stay blind [38]. The incidence of light of these devices in the retina promotes the formation of toxic molecules in the cells (photoreceptors or non-photoreceptors) with a higher risk of macular degeneration.

In this work, we describe the physiopathology and risk factors of the two types of AMD, providing the current state of the art about available treatment approaches and outputs of clinical trials run since 2015.

## 2. Age-Related Macular Degeneration Classification Systems

In order to define AMD progression, several classification systems were created. The international age-related maculopathy epidemiologic study group developed a classification system in which the patients who presented some changes in the macula—for example, slight or moderate nonexudative age-related alterations—were diagnosed as having age-related maculopathy [39]. To establish the diagnosis of dry versus wet AMD, it was necessary to identify advanced RPE geographic atrophy and choroidal neovascularization (CNV), respectively [36].

The Wisconsin Age-Related Maculopathy Grading System created another classification system in which AMD is categorized into two types: the early AMD and the late AMD. In the early AMD, soft indistinct or reticular drusen or hard distinct/soft distinct drusen with pigmentary abnormalities is present. In this case, the RPE suffers depigmentation or there is an increase in the retinal pigment. On the other hand, late AMD was described to show geographic atrophy (dry AMD) or exudative (wet) AMD. In exudative AMD, some exudative lesions are present, such as (i) lesions resulting from prior laser treatment for wet AMD, (ii) pigment epithelial detachment, (iii) retinal detachment (associated with the age), (iv) subretinal hemorrhage, or (v) subretinal scar [40].

Another classification system is based on a 9-step scale to rate the severity of AMD takes into account fundus photographs. This classification system was developed by the Age-Related Eye Disease Study Group. All the patients were categorized based on the presence or absence of some AMD characteristics, such as (i) drusen characteristics, i.e., size, area and shape; (ii) abnormalities on the RPE pigmentary, i.e., hypopigmentation/hyperpigmentation and the geographic atrophy; and (iii) retinal findings, i.e., subretinal scars, detachments or hemorrhage [41]. This classification system considers one risk factor for progressing from early AMD to advanced AMD, which is the existence of drusens with a size of more than 125 microns. Another risk factor is the presence of pigment irregularity. In the case of drusen with intermediate size in both eyes, this also typifies a risk factor. All the risk factors are added between the values obtained for both eyes, and the result is numbered between 0 to 4, where 0 stands for the lowest risk. The score obtained translates the potential risk of developing advanced AMD in approximately 5 years’ time, whereas one factor means a potential risk of 3%, two factors 12%, three factors 25%, and four factors 50% [41]. Another model was established using the data from the Age-Related Eye Disease Study Group but adding other variables, such as genetic, phenotypic, and environmental factors [42]. Then, the scores included the smoking habits, age, family history, and the genetics of the patient. These scores are used to determine a more detailed risk assessment.

To understand the pathophysiology of AMD, it is necessary to know the RPE function, the photoreceptors, and the Bruch’s membrane complex. RPE plays an essential role in maintaining the normal function of the retinal photoreceptor. With aging, RPE cells accumulate intracellular residual bodies, and these bodies have lipofuscin, a brown-yellow age pigment) [43]. The function of the vascular choriocapillaris is to remove the extrude material of RPE cells. However, if the RPE function decreases, there is a change in the permeability of Bruch’s membrane that causes the deposition of the extruded material. Those depositions occur between RPE and the Bruch’s membrane and are responsible for the development of drusen. In patients with AMD, choriocapillaris is thinner; therefore, it decreases the clearance of the material and consequently the formation of drusen [44]. The progression of wet AMD disease is triggered by an inflammatory cascade. The drusen formation may inspire the beginning of the inflammatory cascade. Drusen contain inflammatory components from the cascade pathway and are responsible for photoreceptors loss—i.e., RPE loss. The degeneration of RPE that occurs in AMD leads to a loss of function of Bruch’s membrane. The failure of Bruch’s membrane and increased vascular endothelial growth factors (VEGF) promote the growth of atypical choroidal vessels under the RPE and theoretically under the retina. The newer vessels can bleed and result in the formation of scars. The most severe stage of wet AMD is the development of a disciform spot in the macula that consequently results in a permanent and irreversible loss of central vision [45]. The excessive growth of abnormal bold vessels seen in AMD is a process called choroidal neovascularization (CNV), in which platelet-derived growth factor family, and VEGF sub family play a relevant role. The critical factor for pericytes maintenance is the platelet-derived growth factor (PDGF) that is a mitogen and is expressed in isoforms (PDGF-AA, PDGF-AB, PDGF-BB, PDGF-CC, PDGF-DD) that can bind different receptors [46,47]. PDGF and VEGF have a homologue domain and participate in wet AMD pathogenesis. The PDGF and VEGF levels have been described to appear inversely correlated [46]. The inhibition of PDGF and VEGF families showed stronger antiangiogenic effect when compared with the inhibition of VEGF alone [48]. PDGF is an important proangiogenic stimulus, and consequently, the equilibrium maintenance between the levels of PDGF and VEGF can be essential for managing ocular neovascularization [49]. PDGF is essential in the formation of new vessels and in control of the pericytes action. PDGF is critical in growth and viability of pericytes in order to prevent capillary malformation and retinal neovascularization [50].

The VEGF family is composed of VEGF-A, VEGF-B, VEGF-C, VEGF-D, and VEGF-PlGF and is widely studied in ocular angiogenesis. VEGF-A is a critical factor in neovascularization and the main focus in anti-VEGF therapies [51,52]. The dysregulation of VEGF is the triggered factor for pathological angiogenesis [53]. VEGF stimulates proliferation, tube formation, and the migration and vascular permeability of endothelial cells [54]. In the fetal development, RPE releases VEGF that is essential in the development of choriocapillaris [55]. The release of VEGF from the basal RPE monolayer is essential in the formation of fenestrations in choriocapillaris [56]. Under normal physiological conditions, the ocular VEGF levels are low; however, in pathological conditions, these levels are high in affected zones [53]. Elevated VEGF in RPE promotes the breakdown of barrier integrity and consequently promotes neovascularization [57]. Approaches to target both anti-VEGF and anti-PDGF pathways have been proposed in order to increase the response to therapies [58,59].

The complement system cooperates in innate immune response before the action of macrophages and neutrophils. The complement system is composed of more than 30 proteins and activation products with inflammatory and antimicrobial functions [60]. In AMD pathogenesis, a lack of repression of the complement system occurs [61]. C3, C5b-9, CFH, and CFB are present in drusen and AMD lesions [62]. The dysregulated complement activation and the membrane attack complex in the choriocapillaris contribute to an angiogenic environment that leads to the development of CNV [63].

The angiopoietin-2 plays a role in angiogenesis and in immune activation. Both are critical processes in the pathogenesis of exudative AMD [64]. The levels of this proangiogenic cytokine have been found to be upregulated in the aqueous humor of patients with wet AMD and increase with the severity of the disease [65]. Angiopoietin-2 has become a potential therapeutic in wet AMD due to its role in inflammation. The combination of angiopoietin-2 and VEGF inhibition reduced neovascular lesion formation in chronic CNV in mouse models [66].

Aging increases the risk of AMD, and age-related vascular dysfunction contributes to the progression of AMD. The endothelial dysfunction in the choriocapillaris is important in AMD pathogenesis since the endothelial cells produce a physiological balance in order to regulate the vascular functions [63].

## 3. Exudative Age-Related Macular Degeneration (Wet AMD)

### 3.1. Clinical Presentation

The symptoms in patients with exudative AMD are practically nonexistent, and sometimes only mild symptoms are reported—e.g., loss of central visual acuity, disturbances in contrast or colors, and distorted vision or mild metamorphopsia [67]. The localization of the atrophy is important for rating the gravity of the symptoms. In the macula, the geographic atrophy gives signals of a scotoma, i.e., a blind spot. The blind spot can increase slowly over years and then stabilize [68]. Patients with wet AMD commonly complain about the progressive blurring of their central vision, without pain. This complaint can have an acute or insidious beginning. In the case of hemorrhage in the subretinal space from the CNV, patients report acute start. Patients with choroidal neovascular membranes (CNVM), which are new blood vessels that appear beneath the retina, typically describe insidious blurring attributed to the shallow subretinal fluid or epithelial detachments. The CNVM breakdown the existing barrier in the choroid and retina, and if they bleed, it leads to a vision loss [69]. Additionally, patients with CNVM complain of metamorphopsia, difficulty reading a book, and central scotomas. Most of the reports of wet AMD and sporadically dry AMD show stable central scotoma. The central scotomas result in loss of visual acuity and, consequently, difficulty in performing daily tasks. There are no reports of loss of peripheral visual acuity. AMD has also been associated with neurodegenerative diseases, such as Parkinson and Alzheimer’s diseases [70], regardless of patient lifestyle [71,72].

AMD is asymmetric but appears bilaterally, with a reduction in patients’ visual acuity and with some variability between individuals. The Amsler Grid is a square-shaped grid test used to help monitor and detect metamorphopsia or scotoma that involves the central visual field in some diseases in the macula. Home monitoring uses this test to detect early metamorphopsia in patients with exudative AMD [73]. The CNVM is a critical factor in AMD patients. The patient’s eyes with wet AMD have some characteristics, such as (i) subretinal fluid, (ii) retinal pigment epithelial detachment, (iii) hemorrhage in the subretinal space, (iv) lipid deposition on subretinal space, (v) RPE hypertrophy or (vi) atrophy, and (vii) drusen [74]. The CNV can present a particular discoloration in the subretinal field and is delimited with a pigment ring in some patients. The CNV that appears in the breaks of the Bruch’s membrane and grows under the RPE and the CNV that grows between the RPE and the photoreceptors have clinical manifestations, e.g., RPE detachment and lesions with a gray-green coloration (due to the hyperplastic response of RPE) under the retina with thickening of the retinal tissues [75]. A subretinal hemorrhage is present in both cases [76]. A sub-macular hemorrhage is a common complication that can occur in CNVM secondary to wet AMD [77].

### 3.2. Pharmacological Treatments

In clinical settings, the most commonly used pharmacological classes are the antiangiogenic drugs; however, it is possible to use combination therapies with laser treatment. Pre-clinical and clinical models showed that vascular endothelial growth factor (VEGF) is the gold standard mediator in ocular angiogenesis [78]. Five treatments are available for the inhibition of the VEGF pathway (Table 2). Thus, pharmaceutical formulations with agents that block or neutralize VEGF expression are the main target.

Aflibercept was approved in the United States in November 2011 (Table 2) and in Europe one year after (November 2012). In the clinical trials VIEW 1 (conducted in the United States) and VIEW 2 (conducted in Europe), aflibercept showed non-inferiority after eight weekly treatments when compared with monthly treatment with ranibizumab [79,80].

In the MARINA study for occult choroidal neovascularization, 95% of patients who received 0.5 mg monthly of ranibizumab had increased or stable vision against 64% of the patients who received verteporfin (photodynamic therapy) [81].

The CATT trial (comparison of choroidal neovascularization, in AMD, treatment trials) in the United States compared ranibizumab and bevacizumab. These two drugs used for the treatment of neovascular AMD revealed similar visual acuity outcomes [82].

The safety of anti-VEGF agents in systemic and ocular terms has been a concern to the scientific community. Intravitreal injections are associated with the risk of infection: endophthalmitis by *Streptococcus* and *Staphylococcus* species [83]. For example, in clinical trials, the administration of pegaptanib showed adverse events that required vigilance, among them endophthalmitis, retinal detachment, and traumatic injury of the lens [84]. Ongoing clinical trials for novel anti-angiogenic therapies for AMD are described in Table 3. Several decreases in visual acuity accompanied these adverse effects. Grave uveitis and endophthalmitis occurred in patients with ranibizumab administrations over 24 months [81]. Adverse effects after brolucizumab injections were (i) higher intraocular pressure after 30 min of administration, which is a risk to arterial thromboembolic events, and (ii) intraocular inflammation, (iii) hypersensitivity, (iv) blurred vision, (v) periocular infection, (vi) eye pain, (vii) hemorrhage, and (viii) cataract [85]. Laser treatments approved in AMD include thermal laser photocoagulation, photodynamic therapy, and verteporfin therapy.

The therapies described previously provide stabilization of the disease in wet AMD. However, of the high costs of the treatments results in low patient long-term compliance. To decrease the financial burden of these treatments, biosimilar medications have been used [86]. Biosimilar medications replicate the therapeutic endpoint of pre-existing medications without copying their molecular structure, as occurs in generic medications [87]. There are biosimilar medications of ranibizumab, aflibercept, and bevacizumab used in neovascular AMD [88]. Ranibizumab biosimilars are razumab (approved in 2015), FYB 201 (phase III trial completed), SB-11 (phase III trial completed), Xlucane (phase III trial underway), PF582 (phase I/II trial completed), CHS3551 (pre-clinical investigation) [88,89]. Aflibercept biosimilars are FYB203 (pre-clinical investigation), ALT-L9 (entering phase I trial), CHS-2020 (pre-clinical investigation), and MYL1710 (phase III trial underway). The bevacizumab biosimilar is ONS-5010 (phase III trial completed) [88].

**Table 2:** Antiangiogenic drugs used in the treatment of wet AMD.

Formulation		Administration	Mechanism of Action	Results	Approval	References
Aflibercept	Fusion protein	Intravitreal injection, 2 mg monthly or every 2 months	Connection to all forms of VEGF-A	Safety and tolerability with similar visual results than ranibizumab. Aflibercept enhanced vision, i.e., 96%, 95%, and 95% of patients taking injections of 0.5 mg monthly, 2 mg monthly, and 2 mg every 2 months, respectively.	Approved for the first time in November 2011. August 2018 approval of the administration of Aflibercept every 12 weeks after the first 1 year of effective treatment.	[90]
Bevacizumab	Humanized full length humanized monoclonal antibody	Infusion, 5 mg/kg every 2 weeks for 2–3 treatments	Off-label VEGF inhibitor	Increase in mean visual acuity and central retinal thickness. Bevacizumab had comparable effectiveness compared with ranibizumab on visual acuity after 1 year of treatment.	FDA has not approved bevacizumab for ocular indications.	[91,92]
Brolucizumab	Humanized single-chain antibody fragment (scFv)	Intravitreal injection, 6 mg every month for 3 months, then, some patients were maintained over 2 or 3 months dosing interval	Small molecule with high affinity to all VEGF-A isoforms; inhibition of activation of the receptors of VEGF and consequent avoidance of the ligand-receptor interaction	Compared with aflibercept, showed non inferiority in mean change in best corrected visual acuity in one year. Reduction occurred in central subfield thickness. Few patients had intra-retinal and/or sub-retinal fluid. These fluids are a marker of AMD.	Approved in October 2019.	[93]
Pegaptanib sodium	Polyethylene glycol conjugated oligonucleotide	Intravitreal injection of 0.3 mg every 6 weeks	Connection and neutralization of VEGF165 (principal VEGF isomer in CNV)	Safety and efficacy. Risk of severe loss of visual acuity was reduced from 22% in placebo group to 10% in the group treated with pegaptanib sodium. Patients preserved their visual acuity (33%) or increased acuity (23%).	Food and Drug Administration (FDA) approved in 2004.	[94]
Ranibizumab	Humanized fragmented monoclonal antibody	24 monthly intravitreal injections of 0.3 or 0.5 mg	Connection and inhibition of isoforms of VEGF	After 12 months of accompaniment, 95% of patients had enhanced or stable vision in both dosages. The visual acuity increased 24.8% with 0.3 mg and 33.8% with 0.5 mg. These results were maintained for 24 months.	FDA approved in 2006.	[81]

Medications that target both anti-VEGF and anti-PDGF pathways have also been studied to increase the therapeutic response. These medications are CLS-AX (pre-clinical investigation), pegpleranib (phase III trial completed), rinucumab (phase II trial completed), DE-120 (pre-clinical investigation halted), and X-82 (phase II trial completed) [95,96,97,98].

Gene therapy is also a promising treatment for wet AMD with the purpose of inhibiting and/or changing the pathogenesis of the disease. Some promising products under study are AAV2-sFLT01 (phase I trial completed), AVA-101 (phase II trial completed), ADVM-022/ADVM-032 (phase I trial completed), RGX-214 (phase I/II underway), Retinostat (phase 1 completed), and AAVCAGsCD59 (phase I underway) [99,100,101,102,103,104].

**Table 3:** Examples of ongoing clinical trials for novel anti-angiogenic therapies for AMD. (https://ClinicalTrials.gov) accessed on 4 November 2021.

Title	EudraCT Number	Medical Condition	Sponsor Name	Start Date
Predictability of response of aflibercept treatment for wet age-related macular degeneration under the treat-and-moderate extend regimen (TMER) treatment model	2015-001394-41	Wet AMD	Raimo Tuuminen	2015-06-10
The effect of intravitreal bevacizumab injections in patients with macular oedema caused by AMD, CRVO or DME on the plasticity of nerves studied by visual evoked potentials	2012-000765-20	Wet AMD, diabetic macular edema, central retinal vein occlusion	Kuopion yliopistollinen sairaala/silmätautien poliklinikka	2015-11-27
The effect of intravitreal aflibercept on ocular perfusion—a pilot study	2016-004608-78	Wet AMD	Kepler University Hospital, Institute of Ophthalmology	2017-03-03
Evaluation the pharmacokinetics, safety, tolerability of single intravitreal injection RC28-E in subjects with wet AMD	ClinicalTrials.gov Identifier: NCT03777254	Wet AMD	RemeGen Co., Ltd.	2018-12-17
Safety, tolerability and pharmacodynamics of single rising intravitreal and multiple rising intravitreal doses of BI 836880 in patients with wet AMD (open label, non-randomized, uncontrolled)	2017-001221-40	Wet AMD	Boehringer Ingelheim Pharma GmbH & Co. KG	2019-05-06
Zoledronic acid as adjuvant therapy in neovascular age-related macular degeneration (The Z-AMD study): a randomized controlled pilot study	2019-001492-37	Wet AMD	Oslo University Hospital	2019-11-01
Long-term study of ADVM-022 in neovascular AMD	ClinicalTrials.gov Identifier: NCT04645212	Wet AMD	Adverum Biotechnologies, Inc.	2020-11-27
RGX-314 gene therapy pharmacodynamic study for neovascular AMD	ClinicalTrials.gov Identifier: NCT04832724	Wet AMD	Regenxbio Inc.	2021-02-22
A multicenter, open-label extension study to evaluate the long-term safety and tolerability of faricimab in patients with neovascular age-related macular degeneration	2020-004523-16	Neovascular age-related macular degeneration (nAMD)	F. Hoffmann La-Roche Ltd.	2021-04-12
A phase 3, multicentre, double-masked, randomised study to evaluate the efficacy and safety of intravitreal OPT-302 in combination with aflibercept, compared with aflibercept alone, in participants with wet AMD	2020-004694-46	Wet AMD	Opthea Limited	2021-08-06
A phase 3 open-label, multicenter, extension study to evaluate the long-term safety and efficacy of pegcetacoplan in subjects with geographic atrophy secondary to age-related macular degeneration	2020-002931-32	Geographic atrophy secondary to AMD	Apellis Pharmaceuticals Inc.	2021-09-17
A study to evaluate the efficacy and safety of intravitreal KSI-301 compared with intravitreal aflibercept in participants with neovascular (wet) age-related macular degeneration (DAYLIGHT)	2021-000225-27	Wet AMD	Kodiak Sciences Inc.	2021-10-26

### 3.3. Combination Therapies

Before the advent of anti-VEGF agents, some studies showed that the use of laser photocoagulation of CNV (either extrafoveal, juxtafoveal, or subfoveal) decreased the risk of loss of visual acuity compared with patients without any treatment [105]. For the treatment, patients needed to be eligible according to their type of CNV determined by fluorescein angiography (FA). Only patients with classic CNV were eligible for this treatment. However, only around 26% of them with wet AMD had this CNV pattern [106,107,108]. Before 2006, TLP was used in patients with CNV outside the fovea and usually in the treatment of variants of wet AMD, such as retinal angiomatous proliferation and polypoidal choroidal vasculopathy [109]. The treatment of subfoveal CNV is no longer performed with TLP because it induces immediate and iatrogenic central scotoma [106]. Since 2006, both TLP and dynamic phototherapy have been replaced by anti-angiogenic injections in AMD treatment [110].

TLP was replaced by photodynamic therapy (PDT) to avoid the central blinding scotoma in the subfoveal CNV after TLP treatments [111]. After the administration by intravenous infusion of a photosensitizing dye, its accumulation occurs in the pathological choroidal neovascular tissues. The dye is stimulated by light with a specific wavelength directly in the CNV, which provokes selective destruction of pathologic vessels—i.e., the leaking vessels are sealed, and the healthy ones are preserved. PDT is not a cure—i.e., it will not rebuild the vision already lost; it will just preserve the healthy vessels [112].

Verteporfin (Visudyne™) therapy is PDT with verteporfin drug. This therapy was approved in cases of classic subfoveal CNV in AMD. Verteporfin is a benzoporphyrin derivative monoacid characterized by a peak of absorption near 689 nm. The activation of Visudyne™ occurs with a specific wavelength of light directed in the patient’s eye for 83 s, which stimulates a photodynamic action in the pathologic vessels. This procedure is described as being better than the previous laser treatments [113]. In patients with classic CNV, PDT with Visudyne™ retards or avoids the loss of vision, maintaining almost constant visual acuity and with decreased necessity of repeat treatment sessions [114].

In addition to the treatments described above (antiangiogenic agents and laser treatments), the possibility of combination therapies still exists. In cases of polypoidal vasculopathy or retinal angiomatous proliferation, the administration of anti-VEGF can result in different outcomes. The maturation of CNV makes a difference and may cause failures in the treatment, leaving the membranes less vulnerable to alterations in the VEGF levels [48,115]. The combination of PDT, which is known to increase the expression of VEGF levels, with anti-VEGF drugs can be an advantage for increasing visual acuity and decreasing the need for recurrent treatments [116,117] (Table 4).

Chen et al. (2020) [118] described the advantages in combination therapies of ranibizumab with PDT, such as the increase in visual acuity and decrease in central macular thickness when compared with ranibizumab monotherapy in AMD patients. Existent CNV was blocked by PDT, and ranibizumab injections are capable of resisting VEGF and repressing CNV, and consequently, the clinical efficiency of combined therapies is strengthened [118]. However, other studies also demonstrate no significant difference in visual acuity outcome when comparing ranibizumab monotherapy and combined therapy with PDT for wet AMD [119,120].

The possibility of surgery to remove the CNV membrane was also proposed. A large randomized clinical trial did not show a substantial benefit for surgical excision of the CNV complex by submacular surgery compared with patients who did not undergo surgery [121].

**Table 4:** Clinical trials with combination therapies.

Techniques	Drug	Results	References
PDT (reduced-fluence)	Bevacizumab	Reduction in the number of treatments by half compared with the administration of bevacizumab alone. No substantial changes in visual outcome.	[122]
PDT (standard-fluence)		Same number of treatments in the combination therapy and bevacizumab. Similar visual acuity was the same in both therapies.	[123]
Verteporfin PDT	Ranibizumab	Increased the visual acuity when combined with ranibizumab. No reduction in the number of treatments with combined therapy.	[124]
Stereotactic radiotherapy		Single dose of stereotactic radiotherapy decreased the number of ranibizumab administered in patients with wet AMD. Visual acuity of patients was maintained compared with ranibizumab alone.	[125]

## 4. Nonexudative Age-Related Macular Degeneration (Dry AMD)

Nonexudative age-related macular degeneration or dry AMD is responsible for about 90% of the diagnosed cases of AMD. The nonexudative AMD causes loss of central vision due to retinal atrophy and degeneration of the central retina. Compared with wet AMD, dry AMD has a slower evolution in central visual loss, which can occur over decades [126]. Patients with dry AMD in advanced form can progress to as profound a visual loss as that occurring in patients with wet AMD. In the early form of dry AMD, it is possible to identify drusen with small/intermediate size without vision loss.

In early AMD, it is possible to identify multiple drusen. In intermediate AMD, the drusen can appear confluent with pigment modifications, and pigment accumulation occurs in the posterior pole. In geographic atrophy (GA) AMD, the RPE appears atrophic, and the visualization of the plexus vascular choroidal is easier. In advanced stages of dry AMD, the formation of large zones of atrophy occurs, with seriously vision damages [127].

The Age-Related Eye Disease Study is a classification system used for dry AMD that includes four categories for identifying the different stages of the disease. In category 1, AMD does not exist—i.e., no drusen or drusen with size <63 microns. In category 2, the early AMD presents multiple small and intermediate drusen: one drusen of at least >125 microns or GA that does not involve the center of the fovea. In the last category, the most serious one, advanced AMD is recognized by GA that involves the foveal center [128].

The loss of RPE is related to an intermediate form of dry AMD and loss of overlying layers of the retina that is called atrophy. The intermediate form also includes complaints of difficulty of the patient in adapting to the alteration in light conditions, decreased reading speed, and sensitivity to contrast. To identify the advanced form of dry AMD, a patient must have present atrophy and severe central visual field loss [2]. Regardless of the form of nonexudative AMD, peripheral visual acuity is maintained. GA is when the area of nonexudative AMD grows to larger areas of RPE atrophy. GA is characterized as bilateral without being symmetric and can promote a rapid loss of vision with the development of CNV. The classification system of AMD developed by the Age-Related Eye Disease Study Group and described in Section 2 is essential for identifying the patients who will develop an advanced form of AMD in the future [129]. The score of loss of visual acuity, or loss of visual field and loss of photoreceptors cells, is a consequence of RPE atrophies, and it characterizes nonexudative AMD [41]. In terms of pathophysiology, nonexudative AMD appears to be inherited with autosomal dominant characteristics and can be aggravated due to environmental factors and/or nutrition habits. It is known that homozygous patients with dry AMD for variant of CFH present higher levels of IL-6, IL-18, and TNF-alpha when compared with heterozygous patients or patients without this variant of CFH [24]. CFH is recognized as an inhibitor of activation and an amplification of the complement cascade [130]. The Y402H polymorphism variations in CFH reduce its ability to inhibit the complement cascade, resulting in deregulated activation of the complement cascade, followed by cytolytic and inflammatory responses [23]. C3a is a bioactive fragment released by the complement activation and is a potent inflammatory mediator; if it occurs with a rare genetic variant of C3, the ability to bind CFH is reduced, and this alteration is related to AMD patients [23,131,132].

Due to atrophy or RPE detachment, degeneration of the choroid and retina occurs. Degeneration of the retina happens in the posterior pole. These events characterize dry AMD. The atrophy is, in most of cases, preceded by drusen. The pathway of GA and, consequently, visual loss has been studied. The death of RPE in GA causes the loss of secondary photoreceptors and, consequently, loss of vision over time [133]. In patients with dry AMD, it was found that RPE cells have low levels of DICER1—an enzyme that cleaves RNA—which leads to a decrease in the break of cytoplasmic RNA-Alu molecules. High levels of RNA-Alu molecules promote the activation of inflammatory proteins (such as NLRP3) and then activation of the cascade of molecular responses that cause RPE cell death occurs [134]. In patients with dry AMD, the major complication that can happen is the progression to wet AMD.

### 4.1. Clinical Presentation

The first signs of nonexudative AMD (early AMD) are described as patients having difficulty seeing by night or with a change in light conditions (intensity)—namely, the adaptation is slow when going from bright outdoors to indoor light. Some patients claim to have visual fluctuations, in which some days have improved vision and in which vision is diminished on other days. In visual distortions, the straight lines seem to look broken [135], and patients have difficulty recognizing faces and reading. Some dry AMD patients revealed distortion of vision, known as metamorphopsia with the slow evolution of atrophy. On the other hand, metamorphopsia is rapid onset in patients with exudative AMD [7]. A funduscopic examination is essential for identifying drusen at the beginning of AMD. In the posterior pole there exists an accumulation of pigment since, typically, AMD patients have confluent drusen with changes in pigment. Atrophy of RPE allows the observation of plexus choroidal in the underlayers [136].

With the progression of the disease, dry AMD presents focal agglomeration of atrophy coalescence and large extension of atrophy; thus, vision is severely compromised. AMD patients usually present areas of drusen, atrophy, and RPE mottling. Amsler grid test, slit-lamp biomicroscopy, and FA are some of the procedures used to study the effects of dry AMD. The evaluation of visual acuity and the Amsler grid test is essential in patients with dry AMD [137]. The first sign of dry AMD is the accumulation of basal laminal and basal linear deposits below the RPE layer. This accumulation is irregular and is associated with RPE hyperplasia and migration phenomena (evident by pigment agglomeration). The increase in basal laminal and basal linear deposits can be soft drusen and/or detachments of RPE. With the increase in drusen’s size, the slow death of photoreceptor cells and/or formation of new vessels (development to wet AMD) can happen. In cases of the death of photoreceptor cells, a narrowing of RPE cells can follow, resulting in atrophy [138]. The dry AMD’s final stage is a very slim choroidal layer without small choroidal vessels below the atrophic RPE. The rod–cone layer covering this area is atrophied too. There are signs of degeneration in the central retina layers. This final stage of dry AMD increases gradually and forms the GA [44].

### 4.2. Pharmacological Treatments

There are no successful treatments for the late atrophic form of AMD—i.e., dry AMD. Even focusing on modulating the complement system, the most recent studies yielded negative results [32,139]. The justification for these treatment failures is the same for other degenerative disorders of the central nervous system; since the disease cascade process has already moved to a point without return, it is too late to repair the damage. In dry AMD, the disease cascade’s neural tissue (i.e., retinal photoreceptors) is irreversibly lost. Nowadays, there is no strategy for preventing the further loss of the photoreceptors present in the periphery of the atrophic zones of the macula [2]. The study of complement inhibitors included the development of eculizumab and lampalizumab [140,141]. Lampalizumab in a phase II trial showed a reduction in GA progression, but in phase III trials it did not decrease the GA enlargement [139,141]. The same occurred for eculizumab, which did not show an effect on GA progression [140]. Another study revealed that using a neuroprotective of central nervous system injuries (similar to the mechanism in GA) could be beneficial. Thus, eye drops of tandospirone were tested. Tandospirone is an agonist of serotonin (5-hydroxytryptamine) 1A receptor, but it did not change the evolution of GA [142]. Thus, there are no approved medications for treating patients with dry AMD; however, some studies showed the beneficial effects of antioxidants, such as vitamin A (beta-carotene as a safe source of vitamin A) and E, zinc, and lutein [143,144,145]. The retina is reported to have a mechanism of defense against oxidative stress, which is important in the pathophysiology of AMD and its evolution, and this mechanism involves vitamin C and vitamin E, carotenoids, zeaxanthin, and lutein [146]. Major structural lipids, such as docosahexaenoic acid (DHA), are essential for regulating membrane permeability, and they play an important role in preventing the formation of new vessels [147]. The Age-Related Eye Disease Study Group reported two different studies relative to nutritional supplementation in patients with AMD [148,149]. One of these studies reported the effect of supplementation of vitamins in high doses—namely, 500 mg of vitamin C, 400 IU of vitamin E, 15 mg of beta-carotene and 80 mg of zinc—in patients with dry AMD. The results showed that the supplementation decreased the risk of dry AMD progression to wet AMD compared with the placebo group; however, there was no decrease in the risk of visual acuity loss. Nevertheless, it is essential to note that nutrition supplements should not be used in patients who smoke due to a greater incidence of lung cancer in active smokers who use a high dose of beta-carotene [150]. The second study of the Age-Related Eye Disease Study Group aimed to increase the efficiency obtained with the supplementation in the first study. Thus, new components (e.g., lutein, zeaxanthin, DHA, and docosahexaenoic and eicosapentaenoic acid) were added to the formulation. Beta-carotene was eliminated from the formulation, and the dose of zinc was adjusted to 25 mg. In this case, the efficiency in the AMD progression was not changed compared with the first study of Age-Related Eye Disease Study Group. The formulation recommended nowadays for dry AMD has 400 IU of vitamin E, 500 mg of vitamin C, 10 mg of lutein, 2 mg of zeaxanthin, 2 mg of copper, and 80 mg or 25 mg of zinc (available in both dosages) [151]. Thus, supplementation with these vitamins, associated with balanced dietary habits, showed that it is possible to reduce the risk of developing dry AMD. Another study demonstrated that the Mediterranean diet may also prevent the set-up of AMD [152]. The consumption of green leafy vegetables and baked fresh fish (source of fatty acids) is beneficial in this prevention [153].

As of now, there is no surgical treatment available to patients with dry AMD. These patients were treated with a very light grid laser technology to reduce the drusen [154]. The results of this therapy showed that a light grid laser induced desorption of drusen and consequently visual acuity melioration in a short time. However, light grid laser technology was related to a higher risk of CNV development in the short term. At the end of the study, the visual benefits were not significant compared with the control group, in which patients were not treated with the laser treatment. In the future, some studies may provide a reasonable method for the treatment of dry AMD, such as the use of genetically modified cells since a phase II study revealed an increase in the retinal thickness in a dose-dependent administration and consequently had higher preserved vision [155].

## 5. Retinal Imaging Studies Used in Both Wet and Dry AMD

Retinal images are used to reveal the detailed changes in patients who have AMD. Several techniques can be used to diagnose, monitor, and guide the treatments of AMD. Some of these techniques are described below.

(i) Optical Coherence Tomography (OCT)

In this technique, infrared light is projected into the retina to create a cross-section retinal view displayed on a computer. Basically, after the projection of low coherence laser in the retina, an image resulting from the time delay is recorded, and the backscattered lights create a cross-section of retina layers [156]. Only a few seconds are needed to obtain the images, without any uncomfortable bright light flashes. In patients with exudative AMD, images reveal pockets of fluid in the retina, and this type of test works as a guide in the treatments with injections. In a patient with early dry AMD, OCT also exhibits drusen under the retina, and in cases more advanced—i.e., late dry AMD—it reveals the retinal thinning from the cell death. The time-domain OCT (TD OCT) technique requires longer times to produce an image. The spectral-domain OCT (SD-OCT) needs less time to obtain images, and it has higher density and better image quality. The polarization-sensitive SD-OCT (PS SD-OCT) was developed to overcome the limitations for detecting the RPE conditions, and swept-source OCT (SS-OCT) technique can penetrate deeper in the tissues with short acquisition times when compared with SD-OCT [157,158,159].

The images obtained by OCT are in hyperreflective and hyporeflective bands that characterize the retina layers. Patients with AMD have four hyperreflective bands in their images, which are believed to illustrate the membrane (external limit), the photoreceptor (inner/outer segment), the RPE, and the Bruch’s membrane [160,161].

The images produced by OCT can show the drusen deposits, the subretinal fluid, pseudodrusen, cases of RPE detachment, and CNV—i.e., AMD abnormalities [158]. The presence of pseudodrusen as a hyperreflective deposit beneath the retina in patients with AMD can reveal an increased risk for end-stage AMD, as well as geographic atrophy and wet AMD. OCT is the technique with the highest sensitivity and specificity for detecting pseudodrusen [162,163,164].

In geography atrophy, the images obtained by OCT show progressive loss of retinal bands. This can be related to the enlargement of the atrophic region that is typically associated with the gradual loss of the outer hyperreflective bands and the narrowing of the outer nuclear layer, the membrane of RPE, and the Bruch’s membrane. These findings were the result of a 12-month follow-up of the patients [165,166].

In the presence of neovascularization, it is possible to see the accumulation of fluids in different levels of the retina in the OCT images. The subretinal fluid behaves as an hyporeflective lesion that is present over the RPE and under the retina [158]. If it is present, a dome shaped form at the RPE layer can identify RPE detachment in OCT results [157]. The presence of the outer retinal tubule in OCT is a structural abnormality that seems like a hypo-reflective center surrounded by a hyperreflective border. The presence of this abnormality reveals the degeneration of photoreceptors; thus, there is no exudative activity for neovascularization, and consequently, the treatment of neovascular AMD is not necessary [167].

OCT has as an advantage in the possibility of studying the changes that can occur in the retinal tissues without an invasive procedure and avoiding systemic complications after other invasive procedures (i.e., invasive angiographic imaging). However, OCT has some limitations in the detailing and grading of the CNV. This limitation can be overcome with the resource of other techniques in conjunction with OCT, as is the case of fundus fluorescence angiography or optical coherence tomography angiography [168].

(ii) Retinal Fundus Photography

Retinal fundus photography (FP) is a simple imaging technique that offers retinal images with color and detects AMD. FP technique projects light from a fundus camera in the dilated pupil to illuminate the fundus [156]. FP is a very useful method for diagnosing both exudative and nonexudative AMD. The obtained images reveal various abnormalities containing lipofuscin, such as drusen (a yellow deposit), blood, lipids (that escaped out of abnormal blood vessels in exudative AMD), scar tissue, reticular pseudodrusen, the area where RPE died (RPE atrophy), and CNV [157,169].

In dry AMD, drusen appear as a yellow round lesion in the images obtained by FP. Around the macula, it is possible to see pigmentary deposit, whereas the atrophic RPE has hypopigmentation. Hard drusen appear with a well-defined border yellow deposit and the soft drusen have an ill-defined border. FP allows categorizing drusen, taking into account their size—i.e., small, intermediate, and large, lower than 63 μm, between 63 and 124 μm, and larger than 125 μm, respectively [168,170]. In exudative AMD, FP can easily detect macular detachment and macular edema [36]. The obtained images are however only two dimensional (2D); thus, they lack depth, and the small details are difficulty to detect—for example, in case of abnormalities (i.e., cataract), the resultant images present with lower clarity [169]. If compared with OCT, FP has lower sensitivity to diagnosed CNV [171]. To detect wet AMD, a diagnosis based on FP alone is considered inadequate because it underestimates the existence of CNV. It is possible to improve the accuracy of this technique in combination with other methods. Thus, it is recommended to combine FP with another technique to avoid its limitations [172].

(iii) The Fundus Autofluorescence

Fundus autofluorescence (FAF) is a technique that allows using a defined wavelength light to activate the fundus fluorescent properties without contrast [36,156]. The defined wavelength of light needs to be around 300 to 500 nm. This range excites the lipofuscin (RPE derivative) with an excitation wavelength of 500–700 nm [169]. The autofluorescence is due to the presence of lipofuscin (biretinoid fluorophore). A fundus spectrophotometer, a fundus camera, or a confocal scanning laser ophthalmoscope are typically used for the FAF analysis. The latter reduces the background from other autofluorescence components present in the anterior segment of the eye [36,169,173]. In comparison with FP, FAF has the advantage since it is possible to detect retinal alterations in the early and intermediate stages of AMD that may appear normal due to lower clarity in FP [173]. FAF detects pigmentary alterations, reticular pseudodrusen, and drusen. In the images of FAF, the lipofuscin resultant of RPE deposits have hyperautofluorescence, and the atrophy of RPE is hypoautofluorescence, and if these conditions are coincident, it is possible to predict that the RPE atrophy is increasing, and FAF is useful for monitoring the geographic atrophy progression (the majority of which occurs in the hyperautofluorescence area) [169]. In lesions, hypoautofluorescence may possibly identify hemorrhages, retinal scares, and fibrovascular membranes. The subretinal fluid is hyperautofluorescent [173]. It is possible to identify the development of wet AMD with FAF with the identification of different patterns—i.e., patchy, reticular, and linear patterns. Patchy pattern in FAF (which occurs in 30.4% of the patients) is most related to the development of wet AMD, followed by the other patterns in a lower frequency rate [174,175].

Another technique that uses a wavelength of light is near-infrared autofluorescence (NIA), where diode laser light is used for excitation (787 nm) and the detection of a specific wavelength above 800 nm. NIA uses the properties of the melanin that is a fluorophore of the retina and is located in the RPE cells and in minor quantities in the choroid. Comparing FAF and NIA in patients with dry AMD, both look dark in the atrophic region, but the adjacent area of the atrophic region seems to appear with increased intensity. Some results described that patients have an enhancement of NIA in normal FAF sites that can reveal that there is an increase in the activity of the melanin before the activity of the lipofuscin [176]. On the other hand, in exudative AMD, the images in NIA and FAF appear dark because of the foiled autofluorescence signals due to subretinal fluid, CNV, or hemorrhage. Nevertheless, the FAF technique has better effectiveness compared with NIA in the detailing of exudative activity [176].

(iv) Fundus Fluorescence Angiography

Fundus Fluorescence Angiography (FFA) is an invasive imaging technique that requires an intravenous administration of fluorescent contrast (resorcinolphthalein sodium). Upon intravenous injection of the dye, a series of photographs are taken to allow the permeability and patency of retinal vessels to be seen [177]. In this technique, a camera is used to project light at 485–500 nm that consequently is reflected by retina layers. Fluorescein absorbs some light and re-emits at 520–535 nm, and then this light is captured by a green filter that produces the final image. This technique is the gold standard in neovascular AMD. It is possible to view the structure of blood vessels and their integrity, providing detailed information on the state of CNV—i.e., structural and leakage state. Thus, it is possible to have images of dye in the vessels, and after dye leakage, images of the choroid and retina.

The classification of CNV based on location is very useful in prognosis and treatment. It takes into account the location of [157]: (i) extrafoveal, approximately 200 to 2500 μm from foveal zone; (ii) subfoveal, bellow the center of the foveal avascular zone; (iii) juxtafoveal, up to 199 μm from the center of foveal zone. Disadvantages of this invasive technique include the systemic complications that can occur after the administration of the fluorescent dye. In addition to staining sclera, skin, and urine, it can result in allergic reactions, nausea, vomiting, cardiac disturbance, respiratory difficulties, anaphylaxis, and ultimately death [178].

(v) Indocyanine green angiography

Indocyanine green angiography (IGA) is a technique that uses a contrast with high molecular weight, the indocyanine green dye, that binds the plasma proteins and consequently leaks less when compared with the images obtained by FFA. IGA uses infrared light, 790nm, to obtain deepest penetration to the RPE structure, offering more information about the CNV location when compared with FFA [158]. As occurs in FFA, IGA uses the injection of an intravenous dye, which makes this technique an invasive procedure too. IGA allows images of subretinal fluids, pigment epithelium detachment, and possible hemorrhages to be obtained, which typically affect the obtainment of images with the FFA method. Lesions with hemorrhages appear as a hypercyanescent hot spot with the IGA technique, and then, it is possible to see detailed characteristics of CNV in AMD patients [179].

## 6. Conclusions

Age-related macular degeneration is an ocular disease that affects millions of people worldwide. Over the last years, many advances have been made in the prevention of AMD progression and in the development of treatment options, such as anti-VEGF agents in the treatment of neovascular AMD. In cases of late atrophic form, clinical research is focused on understanding the pathogenesis of progression, making it possible in the future to direct treatments at specific targets and to treat patients in the optimal timing. On the other hand, although dry form does not have a successful treatment approach, it is known that lifestyle modifications, smoking cessation, use of antioxidants, and physical activity can successfully prevent dry AMD. The development of new therapies for controlling neovascular AMD is an important aim in order to avoid the progression of geographic atrophy—i.e., the evolution from dry age-related macular degeneration to the wet form.

## Data Availability

Not applicable.
